# Five-year incidence of Nd:YAG laser capsulotomy and association with in vitro proliferation of lens epithelial cells from individual specimens: a case control study

**DOI:** 10.1186/1471-2415-14-116

**Published:** 2014-10-02

**Authors:** Karin Sundelin, Nawaf Almarzouki, Yalda Soltanpour, Anne Petersen, Madeleine Zetterberg

**Affiliations:** Institute of Neuroscience and Physiology, Section of Clinical Neuroscience and Rehabilitation/Ophthalmology, The Sahlgrenska Academy at the University of Gothenburg, Sahlgrenska University Hospital, Mölndal, SE-431 80 Sweden; Institute of Biomedicine, Department of Medical Chemistry and Cell Biology, Sahlgrenska Academy at the University of Gothenburg, Mölndal, Sweden

**Keywords:** Cataract, Cell culture, Incidence, Intraocular lens, Lens epithelium, Nd:YAG laser capsulotomy, Posterior capsule opacification, Proliferation

## Abstract

**Background:**

The aims of this study were to determine the 5-year incidence of posterior capsule opacification (PCO) requiring Nd:YAG laser capsulotomy in a representative mixed cohort of cataract patients, to determine risk factors for PCO and to investigate possible association with growth of human lens epithelial cells (HLEC) *in vitro.*

**Methods:**

Pieces of the anterior lens capsule and adhering HLEC were obtained at cataract surgery and cultured individually. After one and two weeks respectively, cultured cells were stained with carboxy-fluorescein diacetate succinimidyl ester (CFDA SE), after which image processing software was used to determine the area of the confluent cell layer. The 5-year incidence of Nd:YAG laser capsulotomy in this cohort was determined through medical records and by mail or telephone interviews. For statistic analyses Mann–Whitney U-test, Fisher’s exact test and binary logistic regression were used.

**Results:**

Data on treatment/no treatment for PCO was obtained from 270 patients with a median follow-up time of 57 months (range 50–64 months). The three-year cumulative incidence of PCO was 5.2% and the cumulative 5-year incidence was 11.9%. Patients who had undergone Nd:YAG laser capsulotomy were significantly younger (median 71 years) than patients who did not receive treatment for PCO (median 75 years, p = 0.022). Logistic regression demonstrated that apart from younger age, follow-up time and type of intraocular lens (IOL) were associated with risk of PCO, with hydrophilic 1-piece IOLs conferring a higher risk than hydrophobic acrylic 1-piece or 3-piece IOLs (adjusted OR = 9.4, 95% CI 2.5-35.7, p = 0.001). Of the 270 patients from whom information could be retrieved regarding PCO treatment, *in vitro* cell culture could be established and quantified from 185 patients. No significant difference in cell growth *in vitro* was shown between patients subsequently requiring/not requiring Nd:YAG laser capsulotomy.

**Conclusions:**

The cumulative 5-year incidence of 11.9% is comparable or slightly higher than reported in other recent studies. The type of IOL was the most important risk factor for PCO in this study, whereas intrinsic proliferative capacity of the individual’s lens epithelial cells seems to be less important for subsequent PCO development.

## Background

Although cataract surgery in most patients is a standard procedure with low complication rates, posterior capsule opacification (PCO) remains a relatively common condition postoperatively [1]. In a majority of patients, PCO can easily be treated with neodymium-doped yttrium aluminium garnet (Nd:YAG) laser capsulotomy. However, this treatment can itself result in complications such as raise in intraocular pressure, inflammation, cystoid macular edema or retinal detachment [2, 3]. Many approaches have been tried in order to prevent PCO, both pharmacological or cytotoxic measures to affect lens cell viability, different materials and design of the intraocular lens (IOL) and various surgical strategies [1, 4]. However, so far the only preventive measure to reduce PCO with solid evidence is a square edged design of the IOL [5]. The first studies on PCO incidence with this type of IOL showed promising results and it was generally considered that the problem of PCO was a matter of the past [6, 7]. Since then, studies with longer follow-up have demonstrated that PCO incidence is still high, suggesting that a square edged IOL may delay the onset of PCO but the problem is far from solved [1, 8].

Previous epidemiologic studies have indicated that individual-related factors such as age, gender and ocular comorbidity may influence the risk of PCO [9–11]. However, the relative contribution of such individual-related risk factors to risk of PCO in comparison with IOL- or surgery-related factors is not known. Also, it is not known whether individual-related factors that promote PCO development are due to intrinsic properties of the lens epithelial cells of that individual or if they are due to individual-dependent systemic factors or differences in the ocular milieu surrounding the lens cells *in vivo*. We therefore set up a study investigating the proliferative capacity of individual lens epithelium capsule specimens *in vitro* and compared this to the risk of experiencing Nd:YAG laser posterior capsulotomy during a 5-year follow-up period. In addition, the 5-year incidence of PCO requiring Nd:YAG laser capsulotomy in a representative cohort of cataract patients in a tertiary eye clinic is reported.

## Methods

In 2007, human lens capsule epithelium specimens were collected from 356 patients during cataract surgery at the Eye Clinic, Sahlgrenska University Hospital, Mölndal. The study was approved by The Regional Ethics Committee in Gothenburg, Västra Götaland County, Sweden, and the tenets of the Declaration of Helsinki were followed. All patients gave informed consent. Collection of specimens and subsequent cell culture was performed as previously described [12]. Briefly, the human lens epithelium specimens were immediately after surgery put in Eppendorf tubes with standard culture medium (RPMI-1640) supplemented with 10% fetal calf serum (FSC), 100 U ml^-1^ penicillin, 0.1 mg ml^-1^ streptomycin and 2 mM L-glutamine. After storage in room temperature for up to 24 hours, the lens capsule epithelium specimens were transferred to 24-well culture dishes (TPP, Switzerland) containing freshly prepared cell culture medium supplemented with 5% FCS. No attempts were made to orient the capsules with one specific side downwards, nor to attach the capsules to the plastic bottom. Instead the capsules were allowed to set freely to the bottom of the culture well. The culture dishes were then put into a humidified CO_2_-incubator at 37°C. After one week, a majority of the lens capsule epithelium specimens, which were approximately 5 mm in diameter, had attached to the bottom of the culture wells and lens cells had started to proliferate and to grow outside the capsule. Cell cultures were subsequently incubated with carboxy-fluorescein diacetate succinimidyl ester (CFDA SE) for 15 minutes at room temperature. CFDA SE (Vybrant CFDA SE, Invitrogen, Eugene, OR, USA) is a fluorogenic non-toxic compound that only enters metabolically active cells. Prior to staining, a stock solution of 10 mM CFDA SE was prepared in dimethyl sulfoxide (DMSO). This stock solution was then further diluted to 10 μM in preheated phosphate buffered saline (PBS). After 15 minutes the CFDA SE solution was replaced by freshly prepared cell culture medium with 5% FCS and lens capsule epithelium specimens were photographed using a confocal microscope (Nikon Eclipse TE300 C1), excitation wavelength 492 nm, emission wavelength 517 nm. One week after the first staining procedure, fluorescence had faded and cells were again incubated with CFDA SE and photographed as above. Images were captured with a Nikon D-Eclipse C1 camera and EZ-C1 3.30 Gold as imaging software.

In 2012, five years after collection and cell culture of the lens capsule specimens, medical records were studied in order to establish if the patients had been subjected to Nd:YAG laser capsulotomy due to posterior capsule opacification. Since this procedure is performed in several eye clinics in the Gothenburg area, letters were sent to all patients who had not had Nd:YAG laser capsulotomy at the Sahlgrenska Eye Clinic, inquiring about PCO treatment elsewhere. Patients not responding by mail were also contacted by telephone. An additional ethical permission was obtained from The Regional Ethics Committee in Gothenburg, Västra Götaland County, Sweden, for the follow-up part of the study before contact was taken with any patients. During the five year follow-up period, 51 persons (14.8%) were deceased. No contact was taken with relatives to the deceased persons in order to inquire about Nd:YAG capsulotomy and hence these persons were excluded from the study. In addition, 35 patients could not be reached and were also excluded, leaving a number of 270 patients from whom reliable information about PCO treatment could be obtained. Medical records were examined for data on pseudoexfoliations, uveitis, diabetes, type of IOL and topical anti-inflammatory treatment postoperatively. Several types of IOL were used during the period when lens capsule specimens were collected; Tecnis ZA9003 (Abbott Medical Optics, CA, USA), AcrySof SN60AT, SN60WF and MA60MA (Alcon Inc, TX, USA), Akreos Adapt and Akreos MI60 (Bausch & Lomb, NY, USA), Acri.Smart 36A (Acri.Tec, Carl Zeiss Meditec AG, Germany, recently renamed the CT Asphina 509 M) and Hoya PY-60 AD (Hoya Corporation, Japan). For statistical analysis of the influence of IOL type on PCO incidence, IOLs that were only used in a single patient were not included. The remaining IOLs were grouped accordingly; 1) hydrophobic acrylic 3-piece IOL (Tecnis ZA9003), 2) hydrophobic acrylic 1-piece IOLs (AcrySof SN60AT and SN60WF), 3) hydrophilic acrylic 1-piece IOLs (Akreos Adapt and Akreos MI60). All of these IOLs had a sharp edge design of the optic.

### Statistics

The area of the fluorescent lens capsule epithelium specimens was determined after one and two weeks of culture using image processing software (ImageJ, National Institutes of Health, USA) and the increase in cell layer surface was calculated. Since the increase in cell growth was not normally distributed, Mann–Whitney U-test was used for comparison of cell growth between the group subjected to Nd:YAG laser capsulotomy and the group not requiring PCO treatment. For statistic analysis of demographic characteristics Mann–Whitney U-test and Fisher’s exact test were used. Kaplan-Meier survival plots were constructed for cumulative incidence of eyes not requiring Nd:YAG laser capsulotomy, where patients were censored at the end of follow-up period. Survival plots were also generated after stratification for type of IOL. Binary logistic regression with Nd:YAG capsulotomy as dependent variable was performed in a backward stepwise model with age at cataract surgery, gender, follow-up time, PEX, uveitis, diabetes, type of IOL and topical anti-inflammatory treatment as covariates. Significant parameters, along with age and gender, were then included in the final model. Significance was set at *P* < 0.05. SPSS 16.0 (SPSS Inc, Chicago, Il) was used as statistic software.

## Results

**Figure 1:**
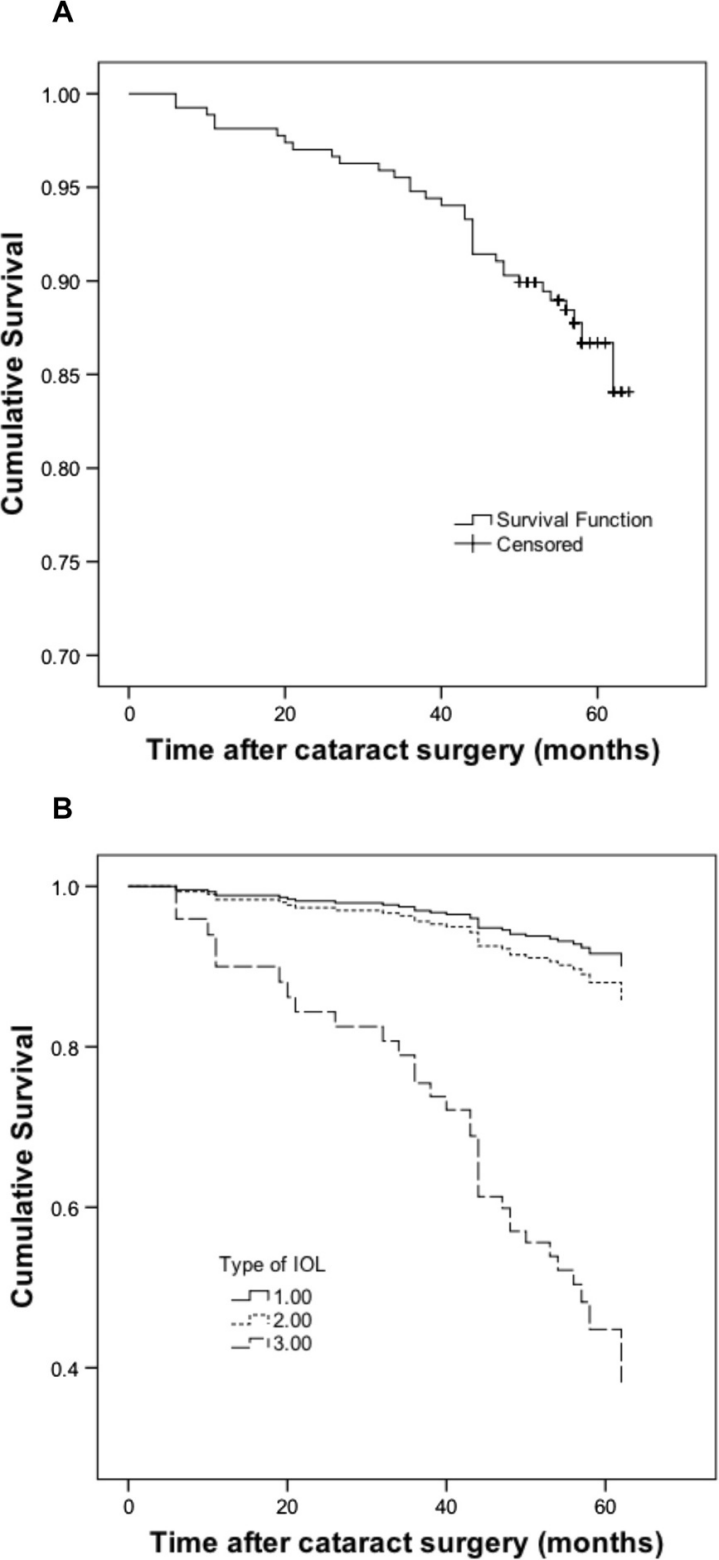
**Kaplan-Meier survival plots of eyes not requiring Nd:YAG laser posterior capsulotomy.** Patients are censored at the end of follow-up. For the whole study group, 268 patients were included at the beginning of the period **(A)**. Patients were also stratified according to IOL type **(B)**. Only IOLs used in more than one patient were included, yielding a number of 264 patients. The IOLs were then subtyped accordingly: 1) hydrophobic acrylic 3-piece IOL (Tecnis ZA9003) n = 93, 2) hydrophobic acrylic 1-piece IOLs (AcrySof SN60AT and SN60WF) n = 157, 3) hydrophilic acrylic 1-piece IOLs (Akreos Adapt and Akreos MI60) n = 14.

**Figure 2:**
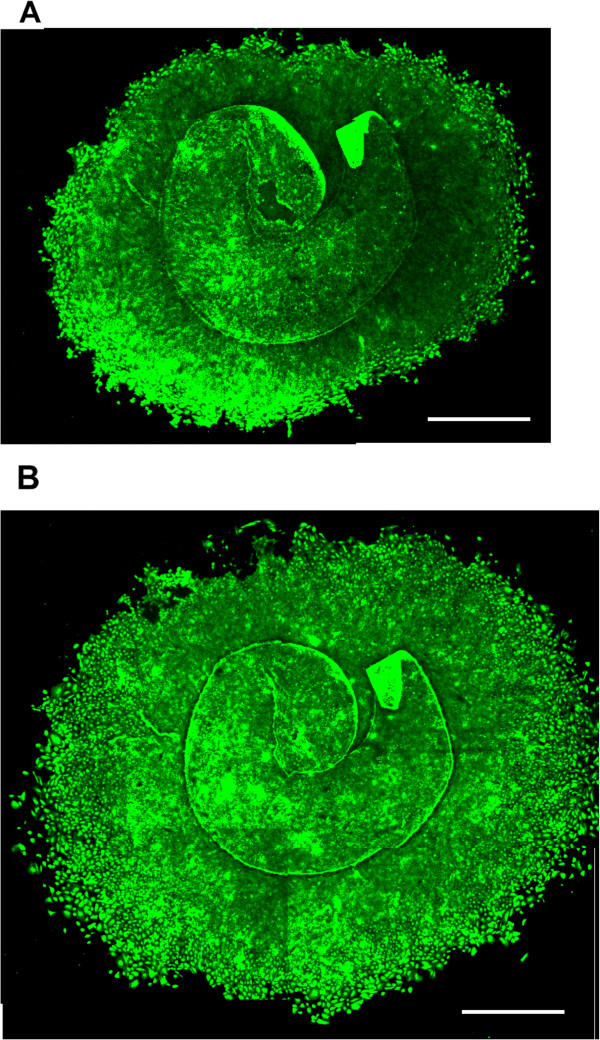
**Growth of human lens epithelium cells from capsulorhexis specimens in culture.** The lens explants were stained with carboxy-fluorescein diacetate succinimidyl ester (CFDA SE) after one **(A)** and two weeks **(B)** of cell culture and photographed with a confocal microscope (Nikon Eclipse TE300 C1), excitation wavelength 492 nm, emission wavelength 517 nm. Scale bar = 2 mm.

**Figure 3:**
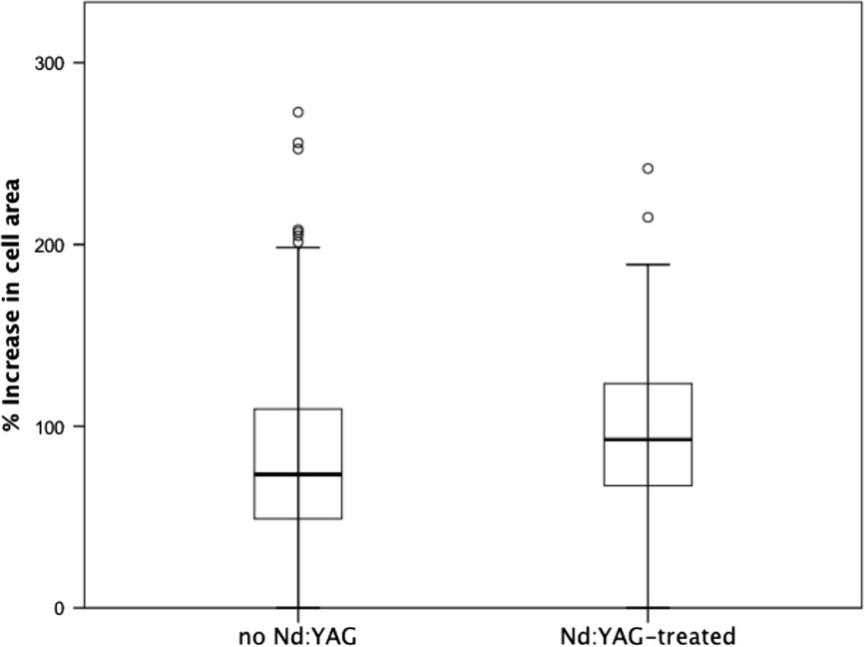
**Growth of human lens epithelium cells in vitro in relation to Nd:YAG-treatment.** Box-plot showing the growth of lens epithelial cells in vitro as % of increase in cell area. Median (bar), interquartile range (boxes) and 95% confidence interval (error bars) as well as outliers are shown. No significant differences between *in vitro* cell growth of lens cells derived from patients later treated with (n = 23) or not treated with Nd:YAG laser capsulotomy (n = 162) was seen (p = 0.24, Mann–Whitney U test).

Medical records or written/oral statements regarding treatment of posterior capsule opacification (PCO) were obtained from 270 patients who had experienced cataract surgery at the Eye Clinic at Sahlgrenska University Hospital, Mölndal, in 2007. Demographic data is presented in Table 1. Patients who had undergone Nd:YAG laser capsulotomy were significantly younger (median 71 years) than patients who did not receive treatment for PCO (median 75 years, p = 0.022). Logistic regression also confirmed that younger age was significantly associated with risk of requiring Nd:YAG laser capsulotomy (adjusted OR = 0.96, 95% CI 0.92-0.99, p = 0.014), see Table 2. Median follow-up time in this study was 57 months (range 50–64 months) and during this period 35 patients (13%) were subjected to Nd:YAG laser capsulotomy. Kaplan-Meier survival plots for eyes not requiring Nd:YAG laser posterior capsulotomy are presented in Figure 1. The three-year cumulative incidence for the whole study group was 5.2% whereas the five-year cumulative incidence was 11.9%. Stratification for type of IOL revealed major differences in Nd:YAG rates (Figure 1B) with the hydrophilic acrylic IOL group having a significantly higher PCO-incidence (adjusted OR = 9.4, 95% CI 2.5-35.7, p = 0.001). Of the 270 patients from whom information could be retrieved regarding PCO treatment, *in vitro* cell culture could be established and quantified from 185 patients. These patients included those where no cell growth could be detected but where the capsule specimen was attached to the culture dish. See Figure 2 for appearance of representative lens capsule explant after one and two weeks of culture. No significant difference in cell growth *in vitro* was seen between patients treated with or not treated with Nd:YAG laser capsulotomy (p = 0.24, Mann–Whitney U test, Figure 3).

**Table 1:** **Demographic and clinical characteristics of cataract patients, N = 270**

Variable	no Nd:YAG	Nd:YAG-treated	***P***-value
	(n = 235)	(n = 35)	
**Age at surgery,** median (10th-90th percentiles), N = 270	75 (58.6-83.0)	71 (44.0-81.4)	0.022*
**Female gender**, n (%), N = 270	157 (66.8)	24 (68.6)	1.00^†^
**Pseudoexfoliations**	48 (21.7)	9 (25.7)	0.66^†^
**present preoperatively**,			
n (%), N = 256			
**Uveitis diagnosis prior to cataract surgery**, n (%), N = 256	7 (3.2)	1 (2.9)	1.00^†^
**Diabetes prior to cataract surgery**, n (%), N = 255	17 (7.7)	5 (14.7)	0.188^†^
**Type of IOL**, n (%), N = 268			
- Tecnis ZA9003	85 (36.5)	8 (22.9)	
- SN60AT	75 (32.2)	8 (22.9)	
- Acrysof SN60WF	64 (27.5)	10 (28.6)	
- MA60MA	1 (0.4)	0 (0.0)	
- Akreos Adapt	2 (0.9)	2 (5.7)	
- Akreos MI60	4 (1.7)	6 (17.1)	
- Acrismart	1 (0.4)	0 (0.0)	
- Hoya PY-60 AD	1 (0.4)	1 (2.9)	
**Additional topical**	20 (8.5)	3 (8.6)	1.00^†^
**anti-inflammatory**			
**treatment postoperatively** ^**‡**^			
n (%), N = 270			

IOL = intraocular lens, Nd:YAG = neodymium-doped yttrium aluminium garnet.

*Mann–Whitney U-test.

^†^Fisher’s exact test.

^**‡**^Topical anti-inflammatory treatment postoperatively in addition to 1% dexamethasone 3 times daily for 3 weeks.

**Table 2:** **Multivariate analysis of possible risk factors for requiring Nd:YAG laser capsulotomy, N = 264**

Variable	Regression coefficient	Standard error	OR	95% CI	***P-***value*
Age^†^	-0.044	0.018	0.957	0.924 – 0.991	0.014
Male gender	-0.274	0.434	0.760	0.325 – 1.780	0.528
Follow-up time	0.153	0.057	1.165	1.042 – 1.303	0.007
Hydrophobic 3-piece IOL^‡^	-0.014	0.479	0.986	0.386 – 2.520	0.977
Hydrophobic 1-piece IOL^#^	0.014	0.479	1.014	0.397 – 2.592	0.977
Hydrophilic 1-piece IOL^||^	2.246	0.679	9.448	2.498 – 35.737	0.001

OR = adjusted odds ratio, CI = confidence interval, IOL = intraocular lens.

*Binary logistic regression with Nd:YAG capsulotomy treatment as dependent variable and age, male gender, follow-time and type of IOL as covariates.

^†^Age at cataract surgery.

^‡^Tecnis ZA9003, (n = 93).

^#^Acrysof SN60WF/SN60AT, (n = 157).

^||^Akreos Adapt/Akreos MI60, (n = 14).

## Discussion

An often cited meta-analysis by Schaumberg et al. from 1998 reported pooled estimates of the proportion of eyes developing visually significant PCO of 11.8% (at 1 year postoperatively), 20.7% (at 3 years) and 28.4% (at 5 years) [13]. However, there was a wide range among published PCO rates; for the 5-year incidence the PCO rate varied between 18.4% and 38.4%. Recent studies have reported incidence numbers of PCO requiring Nd:YAG laser capsulotomy between 2% and 29%, depending on the follow-up period and the type of IOL [8, 14–16]. Although this is slightly lower than the previously reported PCO-rate, development of PCO remains the most common postoperative complication to cataract surgery, affecting a large number of patients.

The overall 3-year and 5-year incidences of PCO in this study were 5.2% and 11.9% respectively. These numbers demonstrate that a large proportion of the patients requiring Nd:YAG laser capsulotomy develop vision-impairing PCO after the first three years, alternatively that they don’t get access to appropriate treatment until after this time period. Either way, it is important to bare in mind when determining PCO incidence or when designing prospective randomized studies with PCO as outcome that a long term follow-up is essential.

A multivariate analysis of possible risk factors for PCO showed that age, follow-up time and type of IOL were significantly associated with risk of requiring Nd:YAG laser capsulotomy. Median age in the group not treated with Nd:YAG laser capsulotomy was 75 years as opposed to 71 years in the group requiring PCO treatment. The increased risk of PCO in younger patients is well known from previous studies, especially in children where special measures have to be taken, *i.e.* posterior capsulorhexis and anterior vitrectomy, to prevent PCO development [17]. However, even for the age group in the present study, ranging from 32 to 90 years with an interquartile range of 65 to 80 years, it is evident that age has a major impact on the risk of experiencing PCO.

In the same multivariate analysis, the risk of requiring Nd:YAG laser capsulotomy showed a strong association with type of IOL (adjusted OR 9.4 [95% CI 2.5-35.7]). The group with hydrophilic 1-piece IOL (Akreos) exhibited a significantly increased risk of developing visually significant PCO with an incidence of 57.1% as compared to 8.6% in the group with hydrophobic 3-piece IOL (Tecnis) and 11.5% in the group with hydrophobic 1-piece IOL (Acrysof). Noteworthy is that all IOLs in these groups had a sharp edge design. The present results are in accordance with a prospective study by Heatley et al. in which patients were randomized to receive either a hydrophobic (Acrysof SN60AT) or a hydrophilic (Centerflex 570H) acrylic 1-piece IOL in the first eye and then received the alternate IOL in the second eye. After one year the area of PCO as assessed from photographs and imaging software was 4.9% in the hydrophobic IOL group and 50.3% in the hydrophilic IOL group [18]. Another study exhibited Nd:YAG rates after two-years follow-up of 10% for a hydrophobic (Acrysof SN60WF) and 42% for a hydrophilic (BL27) acrylic IOL [19].

In this study, the proliferative capacity of lens epithelium capsule specimens from 356 patients was determined using *in vitro* cell culture technique. Of the 270 patients surviving the follow-up period and from whom reliable information regarding treatment with Nd:YAG laser capsulotomy could be obtained, cell growth/or lack of cell growth could be assessed for 185 patient samples. No significant difference in cell growth could be seen between specimens derived from patients who later underwent Nd:YAG laser capsulotomy and from those who didn’t. These findings indicate that the inherent proliferative capacity of lens epithelium cells from the donors is not a major contributor to the risk of PCO development *in vivo*. Instead, cell proliferation leading to visually significant PCO may be more dependent on the local milieu, like the presence of growth factors which in turn may depend on the degree of intraocular inflammation, or on physical factors promoting cell proliferation, adhesion and migration such as the configuration of the capsule, the capsule/IOL interface and the IOL material.

## Conclusions

Individual differences in intrinsic proliferative capacity of the lens epithelium cells is less important for risk of developing PCO than extrinsic factors. Efforts on preventing PCO should therefore continue to focus on the material and design of the IOL or on other mechanical and chemical strategies.
